# Clinical adverse effects of natalizumab: Protocol for a meta-analysis of randomized double-blind placebo-controlled clinical trails

**DOI:** 10.1097/MD.0000000000011507

**Published:** 2018-07-13

**Authors:** Hao Li, Fang-Hong Shi, Shi-Ying Huang, Shun-Guo Zhang, Zhi-Chun Gu, Ji-Fu Wei

**Affiliations:** a Department of Pharmacy, Shanghai Children's Medical Center, Shanghai Jiao Tong University School of Medicine; b Department of Pharmacy, Renji Hospital, School of Medicine, Shanghai Jiao Tong University, Shanghai; c Research Division of Clinical Pharmacology, the First Affiliated Hospital of Nanjing Medical University, Nanjing, Jiangsu, People's Republic of China.

**Keywords:** adverse events, Crohn's disease, meta-analysis, multiple sclerosis, natalizumab

## Abstract

**Background:**

Natalizumab (NAT), a humanized monoclonal antibody, which binds in both α_4_β_1_ integrins and α_4_β_7_ integrins, is approved for the treatment of multiple sclerosis (MS) and Crohn's disease (CD). An uncommon but serious adverse event from NAT treatment is known as progressive multifocal leukoencephalopathy (PML). However, clinical comprehensive safety evidence of NAT is limited.

**Methods:**

We will search Medline, Embase, Cochrane library, and ClinicalTrials.gov website from inception to May 9, 2018. Double-blind, randomized placebo-controlled trials reporting safety data of NAT will be eligible for inclusion. Outcome variables will include adverse events (AEs) varying degrees and AEs occurring in ≥ 5% patients with NAT or placebo. STATA software (version 12, Statacorp, College Station, TX) will be utilized to assess risk of bias and synthesize data. Outcomes will be reported by weight mean difference (WMD), risk ratios (RRs), and their 95% confidence intervals (95% CIs). *I*^*2*^ statistic will be used to evaluate heterogeneity among studies.

**Results:**

This systemic review and meta-analysis will evaluate serious AEs and AEs of NAT as compared to placebo.

**Conclusion:**

Our study will provide a comprehensive picture of AEs of NAT.

## 1 Introduction

Natalizumab (NAT) is a humanized monoclonal antibody, which binds to α_4_β_1_ and α_4_β_7_ integrins, is improved for the treatment of multiple sclerosis (MS) and Crohn's disease (CD).^[1,2]^ The first clinical trial of NAT for treating MS was published in 1999.^[3]^ To data with nearly 20 years of clinical use of NAT, several published randomized, double-blind, placebo-controlled clinical trials have suggested that NAT remains a very effective option for patients with MS.^[4]^ However, a risk of an uncommon but serious adverse event, namely progressive multifocal leukoencephalopathy (PML) in MS patients receiving natalizumab, leads to NAT withdrawal from the market in 2006.^[5]^ NAT was reintroduced to the market later in 2006 after considering its clinical benefits over risks. The most common serious adverse events (AEs) of NAT for patients with MS are relapsing MS, cholelithiasis and the need for rehabilitation therapy.^[6]^ Although efficacy and safety of NAT have been evaluated or are being evaluated in some large-scale, long-term randomized clinical trials. Evidences of reported AEs in clinical trials of NAT are limited. A comprehensive evaluation of safety of NAT is still needed. In this study, we will present an overview of the safety data of NAT therapy in patients with MS or CD by conducting a systemic review and meta-analysis.

## 2 Methods

This systemic review and meta-analysis will be performed by following the principle of PRISMA (Preferred Reporting Items for Systematic Reviews and Meta-Analyses) guidelines and a priori established protocol (PROSPERO: CRD42018095002).^[7]^ Ethical approval is not required because this is a literature-based systemic review and meta-analysis, which will not involve any subject directly.

### 2.1 Literature search strategy and study selection

**Figure 1:**
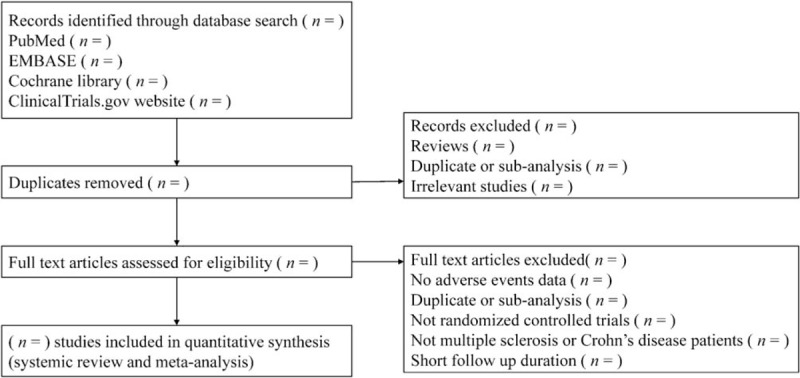
Flow chart of the search process.

We will perform a systemic literature search of relevant databases including Medline, Embase, Cochrane Library, and the ClinicalTrials.gov website from inception to May 31, 2018. The search strategy will be enacted according to the guidance offered from the Cochrane Handbook with the following Medical Subject Heading (MeSH) terms and variants: “natalizumab” or “Tysabri” or “antegren”, and “multiple sclerosis” or “MS” or “Crohn's disease” or “CD” and “clinical trial” or “controlled clinical trial” or “randomized controlled trial” and “placebo” and any possible spellings of “natalizumab” and “multiple sclerosis” and “Crohn's disease”. The search strategy is listed in Table 1. HL and FHS will select and confirm all the publication most relevant to our study including detailed reporting of AEs independently. Disagreements will be resolved by consensus or by consulting a third author (SYH). Literatures that are not conformed to the inclusion criteria or reported incomplete AEs results will be excluded. Details of the selection process are shown in Figure 1.

**Table 1:**
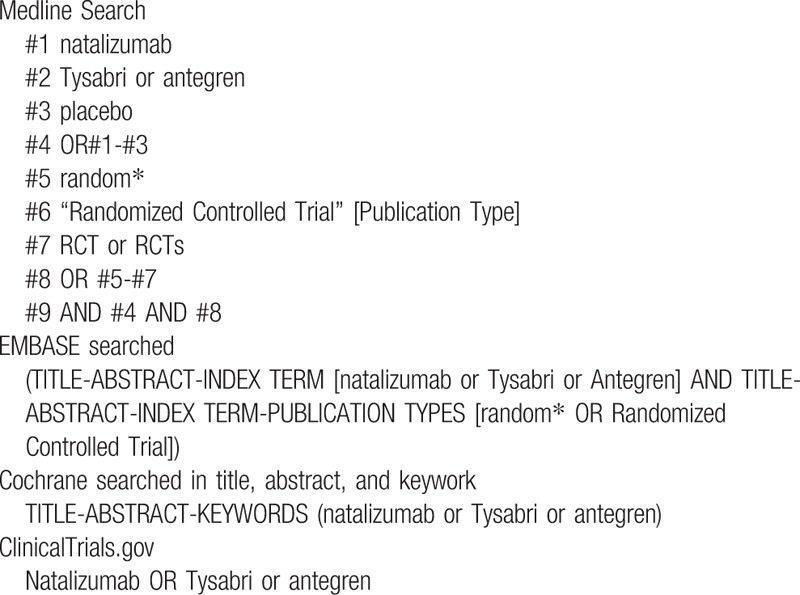
Electronic search strategies.

### 2.2 Outcome measures

Our systemic review and meta-analysis will assess the safety of NAT compared with placebo. For the topic of AEs, we plan to evaluate dosage effect of NAT, AEs varying degrees and AEs occurring in ≥ 5% patients. Another subgroup analysis of AEs will be performed according to disease type (MS and CD), durations of follow up and different dosages of NAT.

### 2.3 Data extraction

The substantial contents of each selected literature will be extracted by HL and FHS. Information should be included these items: first author's name, NCT number, publication time, randomization and control therapies, study duration, study population characteristics (age, sex, duration of disease, renal function, liver function, number of patients), and other details such as different dosages, duration of follow-up, all reported AEs data. Any disagreements will be resolved by consensus or by consulting a third author (SYH).

### 2.4 Quality assessment

Bias risks of studies will be assessed by using the Cochrane tool (Statacorp, College Station, TX).^[8]^ Seven items are related with bias risk, including random sequence generation, allocation concealment, blinding of participants and personnel, blinding of outcome assessment, incomplete outcome data, selective reporting, and other bias, will be assessed by HL and FHS. Low risk, high risk, or unclear risk of bias will be judged and listed. Disagreement of bias risk will be settled by further discussion or consult to the third author (ZCG).

### 2.5 Data synthesis

STATA 12.0 software (version 12, Statacorp, College Station, TX) will be utilized to deal with data extracted from selected articles. Weight mean difference (WMD) and 95% confidence intervals (CI) will be presented in continuous variable and risk ratio (RRs) for dichotomous variable. *I*^*2*^ statistic and *χ*^*2*^ test will be used to evaluate heterogeneity across the studies. The corresponding value lower than 50% will be considered as low heterogeneity, value between 50% and 70% will be considered as moderate heterogeneity, while value above 70% will be considered as high heterogeneity. When *I*^*2*^ is > 50%, a random effects model will be utilized to calculate the effect estimates. While if *I*^*2*^ is < 50%, a fixed effects model will be used. In addition, if quantitative synthesis is no appropriate, qualitative description will be adopted to evaluate the data.

### 2.6 Subgroup analysis

Subgroup analysis based on different dosages of NAT, different type of diseases, durations of treatment, and placebo controls will be conducted.

### 2.7 Sensitivity analysis

Sensitivity analysis will be performed to identify the robustness of the results by omitting each of the study or excluding low-quality studies.

### 2.8 Reporting biases

Funnel plots will be used to evaluate potential reporting biases. Begg test and Egger test will be performed if funnel plots are asymmetry by visual inspection. *P* > .05 in Begg test and Egger test will be considered as no significant publication bias.

### 2.9 Ethics and dissemination

The aims of incoming systemic review and meta-analysis are evaluating current evidence connected with the safety data of NAT for the treatment of MS or CD. No direct subjects will be included and evaluated in this study. Therefore, it does not require ethical assessment in this literature based systemic review and meta-analysis. Results of this incoming study will be disseminated as a literature systemic review and meta-analysis in a peer-reviewed related journal.

## 3 Discussion

MS is a chronic inflammatory disease of the central nervous system, affecting more than 2 million people worldwide with a prevalence of 5 to 30 per million people.^[9,10]^ The relative lack of data from large population countries such as China and India leads to an underestimate of MS.^[10]^ Genetic, environmental, and epigenetic factors drive the condition of MS.^[10]^ Fifteen medications for disease-modifying treatments have been approved by the Food and Drug Administration in the end of 2017.^[9]^ Among these medications, 4 monoclonal antibodies have been approved for MS, which are NAT, alemtuzumab, daclizumab, and ocrelizumab.^[9]^ NAT is the first monoclonal antibody for the treatment of relapsing remitting MS (RRMS).^[10]^ NAT is a nonselective anti-α_4_ integrin monoclonal antibody, binding in both α_4_β_1_ and α_4_β_7_ integrins, could also be utilized in the treatment of CD.^[11]^ CD is a relapsing inflammatory disease, which is a main component of inflammatory bowel disease, affecting the gastrointestinal tract.^[12]^ NAT, opposing the α_4_ chain of α_4_β_7_ integrin and inhibiting the interaction of α_4_β_7_ integrins with endothelial MAdCAM-1 (mucosal addressin cell adhesion molecule-1), leads to a interfere with the homing of lymphocytes to gastrointestinal lymphoid tissue.^[11]^

More than 10 years have passed since the first reports of PML in a patient with MS who were treated with NAT.^[13–15]^ As of December 2017, more than 750 PML cases have been confirmed among patients treated with NAT.^[2]^ There is a fatality rate higher than 20% among PML patients and a substantial morbidity in survivors.^[2]^ Other AEs associated with NAT ranging from serious ones such as hepatic injury, meningitis, and minor ones like headache, hypersensitivity, and so on. The risk of developing opportunistic infections such as meningitis, encephalitis and herpes increases due to an immunomodulation of NAT.^[16]^ Up to now, there is no relevant systematic review and meta-analysis of clinical AEs of NAT.

The purpose of this systemic review and meta-analysis is to assess the safety of NAT in MS or CD patients. We will identify the influence of safety in different dosages of NAT, different diseases, and different follow up durations. Overall, we will give a comprehensive picture of AEs in patients treated with NAT. Different authors will screen articles at least 3 times independently to ensure the accuracy and reliability of the results. Herein, this systemic review and meta-analysis will be the first to evaluate the AEs of NAT in patients treated with NAT, which may offer a comprehensive understanding of NAT.

## Author contributions

HL submitted the registration on PROSPERO. ZCG and JFW are the guarantors for the publication. ZCG take the responsibility for this article. All authors participated in reading and approved the finial article.

**Article revision:** Shi-Ying Huang, Shun-Guo Zhang, Zhi-Chun Gu.

**Conceptualization:** Hao Li, Fang-Hong Shi, Shi-Ying Huang, Shun-Guo Zhang, Zhi-Chun Gu, Ji-Fu Wei.

**Conceptualization:** Hao Li, Fang-Hong Shi, Zhi-Chun Gu, Ji-Fu Wei.

**Data analysis:** Hao Li, Fang-Hong Shi.

**Data curation:** Hao Li, Shi-Ying Huang.

**Study design:** Fang-Hong Shi.

**Study protocol:** Hao Li, Fang-Hong Shi.
